# Polytomous diagnosis of ovarian tumors as benign, borderline, primary invasive or metastatic: development and validation of standard and kernel-based risk prediction models

**DOI:** 10.1186/1471-2288-10-96

**Published:** 2010-10-20

**Authors:** Ben Van Calster, Lil Valentin, Caroline Van Holsbeke, Antonia C Testa, Tom Bourne, Sabine Van Huffel, Dirk Timmerman

**Affiliations:** 1 Department of Electrical Engineering (ESAT-SISTA), Katholieke Universiteit Leuven, Kasteelpark Arenberg 10, B-3001 Leuven, Belgium; 2 Leuvens Kankerinstituut, University Hospitals K.U. Leuven, Herestraat 49, B-3000 Leuven, Belgium; 3 Department of Obstetrics and Gynecology, Skåne University Hospital Malmö, Lund University, SE20502 Malmö, Sweden; 4 Department of Obstetrics and Gynecology, University Hospitals Leuven, Herestraat 49, B-3000 Leuven, Belgium; 5 Department of Obstetrics and Gynecology, Ziekenhuis Oost-Limburg, Schiepse Bos 6, B-3600 Genk, Belgium; 6 Istituto di Clinica Ostetrica e Gynecologica, Università Cattolica del Sacro Cuore, Largo Agostino Gemelli 8, Roma, Italy; 7 Imperial College London, Hammersmith Campus, Du Cane Road, London W12 0HS, UK

## Abstract

**Background:**

Hitherto, risk prediction models for preoperative ultrasound-based diagnosis of ovarian tumors were dichotomous (benign versus malignant). We develop and validate polytomous models (models that predict more than two events) to diagnose ovarian tumors as benign, borderline, primary invasive or metastatic invasive. The main focus is on how different types of models perform and compare.

**Methods:**

A multi-center dataset containing 1066 women was used for model development and internal validation, whilst another multi-center dataset of 1938 women was used for temporal and external validation. Models were based on standard logistic regression and on penalized kernel-based algorithms (least squares support vector machines and kernel logistic regression). We used true polytomous models as well as combinations of dichotomous models based on the 'pairwise coupling' technique to produce polytomous risk estimates. Careful variable selection was performed, based largely on cross-validated *c*-index estimates. Model performance was assessed with the dichotomous *c*-index (i.e. the area under the ROC curve) and a polytomous extension, and with calibration graphs.

**Results:**

For all models, between 9 and 11 predictors were selected. Internal validation was successful with polytomous *c*-indexes between 0.64 and 0.69. For the best model dichotomous *c*-indexes were between 0.73 (primary invasive vs metastatic) and 0.96 (borderline vs metastatic). On temporal and external validation, overall discrimination performance was good with polytomous *c*-indexes between 0.57 and 0.64. However, discrimination between primary and metastatic invasive tumors decreased to near random levels. Standard logistic regression performed well in comparison with advanced algorithms, and combining dichotomous models performed well in comparison with true polytomous models. The best model was a combination of dichotomous logistic regression models. This model is available online.

**Conclusions:**

We have developed models that successfully discriminate between benign, borderline, and invasive ovarian tumors. Methodologically, the combination of dichotomous models was an interesting approach to tackle the polytomous problem. Standard logistic regression models were not outperformed by regularized kernel-based alternatives, a finding to which the careful variable selection procedure will have contributed. The random discrimination between primary and metastatic invasive tumors on temporal/external validation demonstrated once more the necessity of validation studies.

## Background

Medical diagnostic studies typically involve predicting the presence or absence of a target condition. The development of prediction models then becomes a problem of binary classification, even though often multiple differential diagnoses exist. There are statistical and other mathematical techniques to simultaneously predict three or more conditions, but these are underused [1]. Possible reasons for this may be increased complexity, lack of knowledge or lack of data, or the force of habit. If we take as an example the characterization of ovarian tumors, diagnostic models have consistently focused on predicting malignancy versus benignity [2-5]. Notwithstanding the importance of the differentiation between cancerous and non-cancerous tumors, this dichotomization ignores the relevant heterogeneity in malignant tumors. It is known that different types of malignant tumors may be managed differently [6-9], thereby improving the prognosis and reducing unnecessary financial cost or hospitalization. Therefore, in this work, we focus on polytomous risk prediction models to characterize ovarian masses as benign, borderline malignant, primary invasive, or metastatic invasive.

Ovarian cancer is a common and lethal cancer. The American Cancer Society reports that ovarian cancer has the fifth highest death rate of all cancers among females in the United States, with about 15,000 deaths per year [10]. When confronted with an ovarian mass, an accurate preoperative diagnosis is important to decide on the optimal treatment. For benign tumors management may involve a simple "watch and wait" strategy or minimal access surgery. Misdiagnosis of a benign mass as malignant may lead to a woman undergoing a radical surgical procedure for no reason. Hence the consequences of a misclassification may be very serious.

Primary invasive malignancies originate in the ovary, whilst metastatic invasive malignancies originate elsewhere (e.g. breast, colon, stomach, pancreas) but have spread to the adnexal structures. Borderline tumors are of 'low malignant potential', representing less aggressive tumors that are less life-threatening. From a clinical viewpoint differentiating between these different types of tumor has significant relevance. Primary invasive tumors are typically managed using invasive techniques such as laparotomy for staging, interval-debulking surgery or cytoreduction [11]. An intervention for a borderline tumor may be relatively conservative in young women where preservation of fertility is a major issue [9]. For metastatic disease the management option may be influenced by the primary site of malignancy. The differentiation between borderline and invasive tumors is one of the most pertinent clinical issues beyond the differentiation between benignity and malignancy.

In the medical literature, risk prediction models are often based on a logistic regression analysis. In this study, we applied several alternatives to the standard multinomial logistic regression model (MLR) for two reasons. Firstly, in the MLR model the selected variables are used to distinguish between all events whereas variables may be important only for a subset of them [12]. Therefore, we also performed polytomous classification by combining dichotomous logistic regression models to investigate whether this resulted in better performance. Secondly, algorithms more flexible than logistic regression are available, many of them having their origin in the machine learning area. We applied kernel-based polytomous methods based on least squares support vector machines [13] and kernel logistic regression [14]. Another important advantage of these models is that they include a regularization (or penalization) parameter in their standard model formulation to avoid overfitting, a procedure comparable to shrinkage methods for logistic regression models [15]. Because borderline and metastatic tumors had low prevalence such that there was a risk of overfitting, we aimed to compare the performance of these approaches with those based on standard logistic regression analysis (without shrinkage). All algorithms implemented in this study result in probabilities for each of the four events considered.

As explained in the next section, the prediction models were developed and tested (i.e. internally validated) on data from a large international multi-center study. Thorough validation of any developed prediction model is essential to assess the model's robustness and generalizability [16]. Therefore, a temporal and external validation was performed on a large dataset that was collected after model development.

## Methods

### Design and setting

This is an international multi-center cross-sectional study, involving women presenting with an adnexal mass to experienced ultrasound examiners in oncological referral centers, referral centers for ultrasonography, or regional hospitals.

### Data

The International Ovarian Tumor Analysis (IOTA) group [17] collected data from 1066 non-pregnant women with at least one persistent adnexal mass (including para-ovarian and tubal masses). All patients underwent an ultrasound examination by the principal investigator, a gynecologist or radiologist specialized in gynecological ultrasound. Nine clinical centers participated from Italy (4), France (2), Belgium (1), Sweden (1), and the United Kingdom (1). Only patients who were operated on within 120 days after the ultrasound examination were included. The decision whether to operate or not was made by local clinicians based on the clinical picture and local management protocols. More information on inclusion and exclusion criteria is presented in [18]. Data collection was standardized to enhance reproducibility of the measurements [17]. The primary aim of the IOTA study was the development of dichotomous prediction models contrasting benign with malignant tumors [5,18,19].

Data included personal and family history of ovarian and breast cancer, demographic data, grey scale and color Doppler results from ultrasound examination (i.e. more than 40 morphologic and blood flow characteristics describing the tumor), and the presence or absence of pain during examination. After checking for high inter-variable dependencies, 36 variables remained. The outcome of interest was the histological diagnosis of the mass as benign, borderline, primary invasive, or metastatic. Eight hundred tumors were benign (75%), 55 were borderline (5%), 169 were primary invasive (16%), and 42 were metastatic (4%). The dataset was split up in a training set containing 754 patients (71%) for model development, and a test set containing the remaining 312 patients for the internal validation of the models. The split was stratified for center and outcome. We did not use bootstrapping for the internal validation because we did not use fully automated variable selection.

Because the number of borderline and metastatic tumors was limited, we selected 16 variables that were thought to be of potential relevance based on the literature and on subject matter knowledge from clinical experts. This improved the situation, yet the number of borderline and metastatic invasive tumors relative to the number of candidate variables remained small.

After model development, the IOTA group collected a new set of data from 19 centers [20]. Seven centers also contributed to the initial dataset, such that the 941 patients contributed by these centers were used for a temporal validation of the models' performance (654 benign, 69 borderline, 186 primary invasive, 32 metastatic). The 12 centers that did not contribute to the initial dataset were located in Italy (6), Belgium (1), Sweden (1), Poland (1), Czech Republic (1), China (1) and Canada (1). The 997 patients contributed by these 12 centers were used for the external validation of the models (742 benign, 42 borderline, 187 primary invasive, 26 metastatic).

The research protocols for the collection of the development and validation datasets were ratified by the local ethics committee at each recruitment center.

### The kernel-based algorithms

#### Least squares support vector machines

Standard support vector machine (SVM) classifiers [21] are non-probabilistic dichotomous models. First, the predictor space (i.e. the multidimensional scatter plot of the predictors) is mapped into a high dimensional 'feature space'. The aim is to find a feature space where an acceptable linear model can be developed in order to deal with possible nonlinearities in the original predictor space. The linear separation between the events in the feature space tries to maximize the margin between the two groups - hereby imposing regularization - while at the same time controlling the number of misclassifications. A good balance between both is desired: too much focus on margin maximization leads to an overly simplistic model, too much focus on misclassification minimization leads to an overfitted model. A regularization (penalization) parameter is included to control the trade-off. Through the use of a positive definite kernel function it is not necessary to directly work in the high dimensional feature space. The choice of kernel affects how the linear separation in the feature space relates to the predictor space. The linear kernel **x**^*T*^**z **(with **x **and **z **two vectors of predictor values representing two patients) results in linear classifiers in the predictor space whereas other kernels such as the popular Gaussian kernel, $\exp\left(-{\left\|\text{x}-\text{z}\right\|}_{2}^{2}/\sigma^{2}\right)$ with the kernel parameter *σ *that has to be tuned, result in nonlinear classifiers. Least squares SVMs (LS-SVMs) are a variant of SVMs that work much faster due to small changes in the cost function [13]. However, performance of LS-SVMs is similar to that of SVMs [22].

We overcame the non-probabilistic nature of standard (LS-)SVMs through the use of a Bayesian framework [23]. Using the distribution of the outcome in the development data as prior event probabilities, dichotomous event probabilities were obtained based on the LS-SVM output. Hyperparameters such as the regularization and kernel parameters are automatically tuned by the Bayesian procedure.

### Kernel logistic regression (KLR)

In essence, KLR only differs from SVMs with respect to the adopted loss function. However, KLR directly results in probabilistic output and is easily extended to a multinomial version (MKLR). We used an MKLR algorithm that is based on LS-SVMs [14]. The basis of the algorithm is a regularized MLR model that is solved using a penalized negative log likelihood function using iteratively regularized re-weighted least squares. By mapping the predictor space into a high dimensional feature space using a positive definite kernel and applying in each iteration a model with the structure of an LS-SVM, a kernel version of MLR is obtained. The hyperparameters were tuned using five-fold cross-validation (CV).

### True polytomous models versus the combination of dichotomous models

MLR and MKLR simultaneously distinguish between all events. Such 'true' polytomous models are also called all-at-once methods. Other methods combine dichotomous models, and vary with respect to the type of dichotomous models and the method to combine them. When polytomous problems are decomposed, the dichotomous models often contrast each event with all other events (1-versus-all approach) or with each other event (1-versus-1 approach). We favored the 1-versus-1 method for the following reasons. Firstly, from a clinical viewpoint it is interesting to see which variables are useful to discriminate between each pair of events. Secondly, it is mathematically more efficient because lumping together different events in the 1-versus-all approach may result in a complex (i.e. heterogeneous) 'meta-event'. Each dichotomous model was developed on the training cases belonging to the two events involved, and was applied to all test set or validation cases. The probabilities from the 1-versus-1 models were combined to obtain polytomous probabilities (i.e. probabilities for each of the four events that sum to one) using the efficient technique of pairwise coupling [24]. The polytomous probabilities *π_i _*are estimated by solving the following linear system:

$$\pi_{i}=\sum_{j,j\neq i}^{4}{\overset{\wedge }{\pi }}_{ij}\frac{\pi_{i}+\pi_{j}}{k-1},\forall i,\text{with}\sum_{i=1}^{4}\pi_{i}=1\text{ and }\pi_{i}\geq 0.$$

### Variable selection

For logistic regression models, we were careful regarding variable selection due to the small number of borderline and metastatic tumors. We did not use automated procedures based on p-values to directly select a final set of predictors because we prefer variable selection based on criteria similar to the model evaluation criteria, and because automated selection is highly unstable and bound to overfit when there are small groups and a large number of candidate variables [25]. The procedure was as follows. Using stepwise, backward, and manual selection procedures, several possible variable sets were generated. The final set of predictors was selected using three criteria. Two criteria measure information content: the Akaike and the Bayesian Information Criterion (AIC, BIC) [26]. These criteria penalize a model's log likelihood for the number of predictors. AIC has the tendency to be liberal whereas BIC tends to be conservative. Therefore, we prefer models with fairly low values for both criteria. The third and most important criterion was the discrimination performance assessed by the average dichotomous *c*-index for each event after 20 independent runs of stratified five-fold CV. The dichotomous *c*-index equals the area under the ROC curve.

For the kernel-based methods, a forward selection algorithm based on rank-one updates of the kernel matrix in the context of standard LS-SVMs was used [27]. It is computationally intensive to select variables by repeatedly adding the variable that gives the best performance gain based on leave-one-out cross-validation (LOO-CV). When using LS-SVMs, this strategy can be speeded up significantly for two technical reasons. Firstly, the LS-SVM model structure allows for the fast computation of model performance measures based on LOO-CV. Secondly, the LS-SVM model can be updated using rank-one updates in the kernel matrix such that adding a variable does not require the re-computation of the model. This new method, abbreviated as R1U, is very fast, but is currently only available for linear-kernel LS-SVMs. Using the training set, we observed that a linear kernel LS-SVM with R1U-selected variables performed clearly better than linear or nonlinear LS-SVMs using variables that were selected using an advanced nonlinear procedure [28]. In each step, we used the *c*-index estimated by LOO-CV to determine which variable to add. We re-tuned the regularization parameter in each step using a grid search to find the value with maximal *c*-index.

### Overview of methods used to diagnose ovarian tumors

The methods that were used in this study can be divided into two groups based on the variable selection method. The first group consists of two logistic regression-based methods using logistic regression-based variable selection: MLR, and pairwise coupling of 1-versus-1 logistic regression models (LR-PC). The second group consists of three kernel-based methods using variables from R1U selection: MKLR, and pairwise coupling of 1-versus-1 Bayesian LS-SVMs (LSSVM-PC) or KLR models (KLR-PC). We add one logistic regression-based method to the second group for means of comparison: pairwise coupling of 1-versus-1 logistic regression models (LR-PC2).

### Evaluation of model performance

Model training was based on training data only. The models were then applied to the validation datasets for evaluation and comparison. Model evaluation was based primarily on a polytomous extension of the *c*-index. Then the standard *c*-index (i.e. the area under the ROC curve) was used to assess discrimination between every pair of events. The polytomous extension is a measure that bears similarity with the volume under the surface index for trichotomous classification [29]. Assume a set of cases consisting of one case from each event. We defined that perfect separation of the cases in the set was obtained if, for all events, the predicted probability for an event was largest for the case with this particular event [30]. More generally, we counted the number of events for which this was true. For a 4-event problem, with *N*_1 _to *N*_4 _number of cases per event, this polytomous *c*-index was then written as

$$\frac{1}{4N_{1}N_{2}N_{3}N_{4}}\sum_{n_{1}=1}^{N_{1}}\sum_{n_{2}=1}^{N_{2}}\sum_{n_{3}=1}^{N_{3}}\sum_{n_{4}=1}^{N_{4}}C(n_{1},n_{2},n_{3},n_{4}),$$

with *C*(*n*_1_, *n *_2_, *n *_3_, *n *_4_) the number of events for which it held that the event's predicted probability was largest for the case with that event. Thus *C *ranged between 0 and 4. The average *C *over all sets was divided by 4 to obtain a value between 0 and 1 (with 0.25 for random discrimination). This polytomous index can be interpreted as the probability to correctly identify a case from a randomly chosen event within a set of 4 cases.

Calibration was assessed using calibration graphs that related predicted probabilities to actual probabilities using loess smoothing [31,32]. This resulted in four graphs, one per event.

## Results

### Variable selection results

The variables selected for the MLR model and for each dichotomous LR problem are presented in Table 1. Nine variables were selected for MLR. For LR-PC, however, the situation was more complicated. In order to keep the total number of selected variables for the six dichotomous models under control, selection for these models was intertwined. We finally selected three to five variables for each dichotomous problem such that LR-PC used ten variables in total. A similar issue held for the R1U selection. Here, for each dichotomy the variables were ranked based on the LOO *c*-index. Then, starting from these rankings, 20 runs of five-fold CV on the *c*-index were used to select a final variable set per dichotomy while controlling the total number of selected variables. This extensive variable selection strategy resulted in the selection of three to five variables per dichotomous problem, summing up to 11 variables in total for LSSVM-PC, KLR-PC, and LR-PC2 (Table 1). For MKLR, all these 11 variables were used. Thus, the number of selected variables varies between nine and eleven, which is similar to the number of selected variables for the benign-versus-malignant models [5,18,19]. Descriptive statistics for the selected variables are presented in Table 2.

**Table 1:** Overview of selected variables

		Dichotomous 1-versus-1 models					
**Variable**	**MLR**	**Ben vs Bord**	**Ben vs** **PrInv**	**Ben vs Meta**	**Bord** **vs** **PrInv**	**Bord** **vs** **Meta**	**PrInv** **vs** **Meta**

*Logistic regression-based variable selection*							

Ascites	×		×	×	×	×	×

Maximal diameter of solid part	×		×	×	×	×	

Age	×		×	×			

Entirely solid tumor	×			×		×	×

Irregular internal cyst walls	×		×	×			

Personal history of ovarian cancer	×						×

Bilateral tumors	×				×		

Maximal diameter of lesion	×	×					

Papillary structures with blood flow	×	×	×				

Unilocular tumor		×					

							

R1U variable selection*							

Ascites			×	×	×	×	×

Maximal diameter of solid part			×	×	×	×	

Age		×	×		×		

Entirely solid tumor				×		×	×

Irregular internal cyst walls			×	×	×		

Personal history of ovarian cancer		×					×

Bilateral tumors					×		

Maximal diameter of lesion		×					

Papillary structures with blood flow		×					

Number of papillations		×					

Acoustic shadows			×				

Ben: benign; PrInv: primary invasive; Bord: borderline; Meta: metastatic.

* R1U variable selection: variable selection within the framework of least squares support vector machines that is based on rank 1 updates of the kernel matrix [27].

**Table 2:** Descriptive statistics of selected variables for the training data set

	Benign	Border-line	Primary invasive	Meta-static
**Variables**	*N *= 563	*N *= 40	*N *= 121	*N *= 30

**Continuous**				

Age, years (median)	42	52.5	58	59

Maximum diameter of mass, mm (median)	63	108	98	73

Maximum diameter of solid part, mm (median)	0	22	51	54

**Ordinal**				

Number of papillations (mean)#	0.35	1.70	1.43	0.93

**Binary**				

Ascites (%)	3.2	12.5	50.4	40.0

Entirely solid tumor (%)	6.6	7.5	32.2	56.7

Irregular internal cyst walls (%)	33.6	67.5	88.4	83.3

Personal history of ovarian cancer (%)	0.9	5.0	0.8	10.0

Bilateral tumors (%)	17.6	12.5	41.3	33.3

Papillary structures with blood flow (%)	6.8	47.5	43.0	23.3

Acoustic shadows (%)	13.0	2.5	0.0	3.3

Unilocular tumor without solid component (%)	40.3	2.5	0.0	0.0

# Values are 0, 1, 2, 3, 4 (more than three).

### Internal validation (test set results)

On the test set, the polytomous *c*-index varied between 0.64 for the two true polytomous models (MLR and MKLR) and 0.69 for LR-PC2 (Table 3). Thus, for a randomly chosen event, there is a 69% chance that the probability from LR-PC2 for this event is highest for a case with this event than for a case with any of the other three events. Table 4 presents pairwise *c*-indexes for the best models. These results show that pairwise discrimination was generally very good, with the lowest *c*-index for the discrimination between primary invasive and metastatic invasive tumors (*c *0.73). There were very few borderline and metastatic tumors in the test set, which hampered reliable conclusions. Calibration was good, except that the risk of a borderline tumor was overestimated (cf. infra).

**Table 3:** Validation results using a polytomous *c*-index

	Internal validation (n = 312)		Temporal validation (n = 941)		External validation (n = 997)	
**Model** **(# predictors)**	**Polytomous** ***c*-index** **(95% CI)**	**Difference with** **best model** **(95% CI)**	**Polytomous** ***c*-index** **(95% CI)**	**Difference with** **best model** **(95% CI)**	**Polytomous** ***c*-index** **(95% CI)**	**Difference with** **best model (95% CI)**

*Group 1: logistic regression models*						

LR-PC (10)	.67 (.58-.75)	-	.60 (.56-.65)	-	.60 (.55-.65)	-

MLR (9)	.64 (.56-.73)	.025 (-.004; .053)	.58 (.54-.62)	.020 (.000; .040)	.58 (.53-.62)	.028 (.000; .058)

						

Group 2: kernel-based and logistic regression models (based on R1U variable selection)*						

LR-PC2 (11)	.69 (.60-.77)	-	.59 (.55-.64)	-	.64 (.59-.68)	-

KLR-PC (11)	.67 (.59-.75)	.016 (-.013; .051)	.58 (.54-.63)	.012 (-.006; .027)	.61 (.57-.66)	.026 (.004; .049)

LSSVM-PC (11)	.66 (.58-.75)	.025 (-.007; .060)	.58 (.54-.62)	.015 (-.005; .035)	.61 (.57-.65)	.028 (.005; .052)

MKLR (11)	.64 (.56-.73)	.046 (.003; .086)	.57 (.52-.62)	.027 (.000; .056)	.58 (.53-.62)	.060 (.033; .092)

Models are ranked by the value of the polytomous *c*-index. Ben: benign; PrInv: primary invasive; Bord: borderline; Meta: metastatic; CI: confidence interval. 95% CIs are computed using the bias-corrected bootstrap method using 1000 bootstrap samples.

* R1U variable selection: variable selection within the framework of least squares support vector machines that is based on rank 1 updates of the kernel matrix [27].

**Table 4:** Validation results using pairwise *c*-indexes

Model	Ben vs Bord	Ben vs PrInv	Ben vs Meta	Bord vs PrInv	Bord vs Meta	PrInv vs Meta
*Group 1: logistic regression models*						

Internal: LR-PC	.82	.95	.93	.88	.96	.73

Temporal: LR-PC	.88	.95	.93	.81	.83	.51

External: LR-PC	.88	.96	.93	.81	.89	.56

						

Group 2: kernel-based and logistic regression models (based on R1U variable selection)*						

Internal: LR-PC2	.86	.94	.92	.88	.96	.73

Temporal: LR-PC2	.90	.94	.92	.81	.83	.51

External: LR-PC2	.91	.95	.93	.81	.89	.56

Models are ranked by the value of the polytomous *c*-index. Ben: benign; PrInv: primary invasive; Bord: borderline; Meta: metastatic; CI: confidence interval. 95% CIs are computed using the bias-corrected bootstrap method using 1000 bootstrap samples.

* R1U variable selection: variable selection within the framework of least squares support vector machines that is based on rank 1 updates of the kernel matrix [27].

### Temporal and external validation

When aiming to implement a model into clinical practice, good temporal and external validation results are essential. The results that we obtained are presented in Table 3, and show a performance decrease relative to the internal validation. On temporal validation the polytomous *c*-index varied between 0.57 for MKLR and 0.60 for LR-PC, on external validation between 0.58 for MLR and MKLR and 0.64 for LR-PC2. Similar to the internal validation, LR-PC2 and LR-PC were the best models. The pairwise *c*-indexes for these models (Table 4) show that pairwise discrimination among the three malignant events clearly dropped on temporal and external validation. Most striking is the observation that discrimination between primary invasive and metastatic invasive tumors, which was acceptable on internal validation (*c *0.73), was random on temporal (*c *0.51) and external (*c *0.56) validation. Discrimination between these two tumor types and borderline tumors was still good with *c*-indexes above 0.8. Benign tumors can be very well separated from any malignant tumor type, even from borderline tumors (*c*-indexes 0.9 or higher).

The temporal and external validation showed that pairwise coupling of dichotomous models resulted in models with superior discrimination compared to true polytomous models. Also, logistic regression-based models produced better results than KLR or LS-SVM-based models. Taken together, LR-PC2 produced the best results. Calibration was clearly poorer on temporal and external validation than on internal validation (cf. infra).

### Further results for LR-PC2

**Figure 1:**
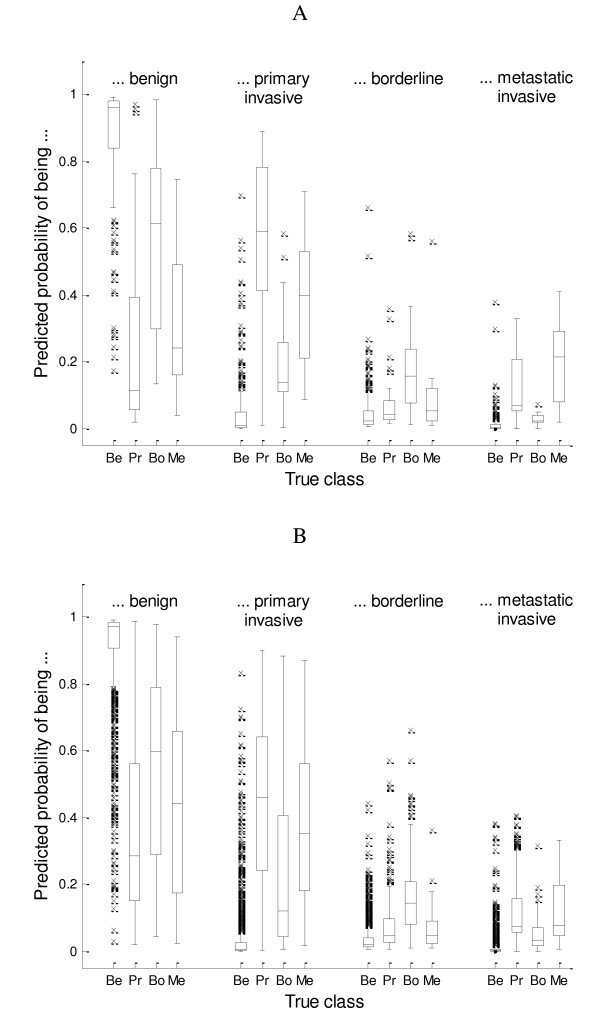
**Box plots of predicted probabilities given by model LR-PC2**. Panel A displays results for the internal validation data, panel B displays results for the aggregated temporal and external validation data. Be: Benign; Pr: Primary invasive; Bo: Borderline; Me: Metastatic.

Figure 1 shows box plots of LR-PC2's predicted probabilities for each event, once for the internal validation data and once for the merged temporal and external validation data. This figure shows that the predicted probability of a specific event is on average higher for tumors with that event compared to tumors with another event. The box plots also show that borderline tumors typically get a high probability for being benign, and that it is less straightforward to separate primary invasive tumors from metastatic invasive tumors. The box plots for the temporal plus external validation data show that the two types of invasive tumors were not distinguishable at all, contrary to what the internal validation box plots suggested.

**Figure 2:**
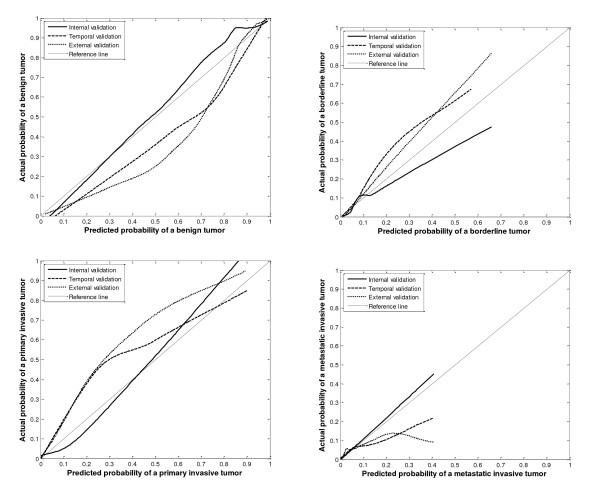
**Calibration graphs for the internal, temporal, and external validation of LR-PC2**.

Calibration was better in probability ranges that contained many patients (i.e. predicted probability of benign tumor of 0.90 or more, probabilities of malignant events up to 0.05-0.10) compared to other regions. The calibration curves are shown in Figure 2. On temporal and external validation, LR-PC2 overestimates the probability of a benign and a metastatic tumor, but underestimates the probability of a borderline and a primary invasive tumor.

We addressed two important clinical issues. The first issue is the role of tumor stage for the discrimination between different tumor types [33]. Primary invasive tumors can be well separated from benign and borderline tumors, but it is of interest to look at primary invasive stage I tumors and primary invasive stage II-IV tumors separately. We focused on the aggregated temporal and external validation data, and computed the pairwise *c*-indexes for the two primary invasive subgroups when compared with benign, borderline, and metastatic tumors. In the development data 32% of the primary invasive tumors were stage I, 7% stage II, 50% stage III, and 11% stage IV. In the temporal/external validation data, primary invasive tumors were 25% stage I, 9% stage II, 57% stage III, and 9% stage IV. Discrimination from benign tumors was very high for both primary invasive subgroups (*c *0.92 for stage I, *c *0.95 for stage II-IV). Discrimination from borderline tumors was clearly poorer for primary invasive stage I tumors (*c *0.70) compared to primary invasive stage II-IV tumors (*c *0.85). Discrimination from metastatic tumors was poor irrespective of stage (*c *0.56 for stage I, *c *0.53 for stage II-IV). The second important clinical issue is ascites. The presence of ascites makes a diagnosis of primary invasive cancer highly likely. When the temporal and external validation data were aggregated, 71% of the patients with ascites had a primary invasive tumor, 13% had a metastatic tumor, and 16% had a benign or borderline tumor. However, it is important that prediction models work well also in patients without ascites. In this subgroup of patients, when combining the temporal and external validation datasets, pairwise *c*-indexes were 0.91 for discriminating benign from borderline tumors, 0.93 for discriminating benign from primary invasive tumors, 0.91 for discriminating benign from metastatic tumors, 0.77 for discriminating borderline from primary invasive tumors, 0.82 for discriminating borderline from metastatic tumors, and 0.57 for discriminating primary invasive from metastatic tumors. The polytomous *c*-index was 0.59. This means that the LR-PC2 performed well also in patients without ascites.

Finally, LR-PC2 can be directly compared with existing dichotomous models using the probability of a benign tumor to discriminate between benign and malignant tumors. LR-PC2 obtained *c*-indexes of 0.939 and 0.954 on temporal and external validation, results that are similar to the main dichotomous model from the IOTA group with *c*-indexes of 0.945 and 0.956 on the same datasets [20].

On http://homes.esat.kuleuven.be/~biomed/LRPC2/lrpc2.htm we have made available an Excel sheet that can be used to implement LR-PC2.

### Comparison of LR-PC2 with a model based on automatic stepwise variable selection

Variable selection for our models was partly based on automatic procedures and partly on human interference. We considered this appropriate, in particular for a model such as LR-PC2 where six dichotomous models with separate variable selection are combined. Therefore, it was interesting to compare LR-PC and LR-PC2 with a similar model based on fully automatic variable selection. We used standard forward stepwise selection for each dichotomous model with p-value criteria for variable entry and removal set at 0.05. The resulting stepLR-PC model used 15 variables in total compared to 10 for LR-PC and 11 for LR-PC2. The polytomous *c*-indexes of stepLR-PC were 0.64 on internal validation (versus 0.67 and 0.69 for LR-PC and LR-PC2), 0.60 on temporal validation (versus 0.60 and 0.59), and 0.58 on external validation (versus 0.60 and 0.64).

## Discussion

Methods for polytomous classification are underused in medical applications. In this paper, we used various methods for the probabilistic diagnosis of ovarian tumors as benign, borderline, primary invasive, or metastatic invasive. To the best of our knowledge, this is the first time that prediction models for ovarian tumor diagnosis exceeded the basic differentiation between benign and malignant tumors. Methods included true polytomous (all-at-once) algorithms and algorithms that combined dichotomous (1-versus-1) models using the technique of pairwise coupling. The basic classification algorithms were based on logistic regression, LS-SVMs, and kernel logistic regression. All models were internally, temporally, and externally validated. Despite the low number of borderline and metastatic tumors, interesting and consistent results were obtained.

The results showed very good separation of benign, borderline, and invasive tumors. This is an important result because the ability to differentiate between borderline and invasive tumors gives additional, highly useful information for making sensible treatment decisions. There is a clear difference in aggressiveness between borderline and invasive tumors, and they are treated differently. The use of a model such as LR-PC2 in clinical practice would therefore be interesting. On http://homes.esat.kuleuven.be/~biomed/LRPC2/lrpc2.htm we have made available an Excel sheet that can be used to implement LR-PC2. Unfortunately, the models were unable to reliably discriminate between primary and metastatic invasive tumors.

In the present study, the combination of 1-versus-1 models with pairwise coupling was an interesting alternative to true polytomous algorithms. The former approach allowed for more fine-tuned variable selection, and resulted in higher validation performance - as determined by the polytomous *c*-index - for both logistic regression-based and kernel logistic regression-based models. An advantage of 1-versus-1 models is their increased flexibility by addressing subproblems that are sometimes of particular interest to the clinician, for example when the clinician hesitates between two diagnoses only. The overall best model combined 1-versus-1 logistic regression models using pairwise coupling (LR-PC2). For the discrimination between benign and malignant tumors (cf. the *c*-index for benign versus other tumors in Tables 3 and 4), this model performed similar to the dichotomous models developed and validated on the same data [18,20]. LR-PC2 used 11 predictors, but not subjective variables such as the experience of abdominal or pelvic pain during the ultrasound examination or the color score of intratumoral blood flow (a subjective score between 1 and 4). These variables are used in some of the existing dichotomous models [18,20]. None of the polytomous models used the CA-125 tumor marker, because this marker was deliberately not considered as a predictor. The most important reasons are that we focused on ultrasound information, and that its use would preclude the immediate use of a model as the results of the blood test have to be awaited. In addition, the inclusion of CA-125 as a variable in dichotomous models did not result in better performance [34].

A disadvantage of combining 1-versus-1 models is that the number of 1-versus-1 problems grows exponentially with the total number of events. Another decomposition of a polytomous problem that consists of a tree of nested (or sequential) dichotomous models does not suffer from this limitation [35,36]. A sensible tree in our study would be to make a model to discriminate between benign and malignant tumors, followed by a model to discriminate between borderline and invasive tumors, and finally a model to contrast primary with metastatic invasive tumors. Polytomous probabilities can be obtained in a straightforward manner. When we applied this approach, it resulted in stronger performance degeneration on temporal and external validation than the true polytomous models or the pairwise coupling approach.

Interestingly, we found that approaches based on logistic regression performed very well when compared to the regularized kernel-based alternatives despite the fact that two events (borderline, metastatic) had very few cases. All models suffered from performance decrease on temporal and external validation, but the decrease was not more severe for the unregularized logistic regression-based models. This might be explained by the careful variable selection strategies for which cross-validated *c*-index estimates were the most important criterion. If we applied pairwise coupling of 1-versus-1 logistic regression models based on standard stepwise variable selection with a p-value of 0.05 as the selection and removal threshold, we ended up with a total of 15 selected variables. The polytomous and pairwise *c*-indexes of stepLR-PC were similar to or worse than those of LR-PC2 and showed stronger decrease on temporal and external validation. That being said, the use of logistic regression models in situations like the one in this study asks for a regularized fitting approach such as shrinkage, penalized maximum likelihood estimation, or the LASSO (least absolute selection and shrinkage operator) [37].

This study further demonstrated the necessity of a thorough validation of prediction models, in particular in situations with small sample sizes for some events relative to the number of variables considered as possible predictors. We observed in our study that, opposite to what the internal validation results suggested, the differentiation between primary and metastatic invasive tumors was near random on temporal and external validation. Unfortunately, many models are developed yet only a limited portion of these undergo validation in various clinical settings. This hampers the successful implementation of such models into clinical practice [38].

Even though the use of LR-PC2 in clinical practice would provide useful information, future work will focus on the development of a more robust model by combining all the data used in this study to update LR-PC2. Ample attention will be devoted to the selection of a limited set of predictors to boost the user-friendliness of the model for busy clinicians.

## Conclusions

This study shows that polytomous discrimination of ovarian tumors can be obtained, while maintaining similar performance for the traditional dichotomous diagnosis (benign vs malignant) and without the need for more predictors. Such models can provide highly useful information for clinicians when having to make sensible treatment decisions. For polytomous prediction, the combination of dichotomous 1-versus-1 models is an interesting alternative to true polytomous (all-at-once) models. Despite two events (borderline, metastatic) with relatively few cases, standard logistic regression approaches performed similar to or better than regularized kernel-based alternatives, a finding to which the careful variable selection based on cross-validated *c*-index estimates will have contributed. The importance of model validation studies is clearly demonstrated as the lack of discrimination between primary invasive and metastatic invasive tumors became clear only on temporal and external validation. Without thorough evaluation of diagnostic performance, it is unsafe to implement prediction models in clinical practice for decision support.

## Abbreviations

AIC: Akaike information criterion; BIC: Schwarz' Bayesian information criterion; CV: Cross-validation; IOTA:International Ovarian Tumor Analysis; KLR: Kernel logistic regression; LOO: Leave-one-out; LR: Logistic regression; LS-SVM: Least squares support vector machines; MKLR: multi-class (polytomous) kernel logistic regression; MLR: Multinomial logistic regression; PC: Pairwise coupling; RBF: Radial basis function; ROC: Receiver operating characteristic; R1U: Variable selection using rank-one updates of the LS-SVM kernel matrix; SVM: Support vector machine

## Competing interests

The authors declare that they have no competing interests.

## Authors' contributions

BVC conceived of the study, participated in its design, performed the statistical analysis, interpreted the results and drafted the manuscript. LV conceived of the study, collected data, interpreted the results, and helped to draft the manuscript. CVH and ACT collected data, interpreted the results, and helped to draft the manuscript. TB conceived of the study, interpreted the results, and helped to draft the manuscript. SVH participated in the study design and analysis, interpreted the results, and helped to draft the manuscript. DT conceived of the study, collected data, interpreted the results, and helped to draft the manuscript. All authors read and improved the final manuscript.

## Pre-publication history

The pre-publication history for this paper can be accessed here:

http://www.biomedcentral.com/1471-2288/10/96/prepub
